# Effectiveness of a clinical decision support algorithm (CDSA) on reducing unnecessary antibiotic prescriptions for upper respiratory tract infections among ambulatory HIV-infected adults in Mozambique: a cluster randomized controlled trial

**DOI:** 10.21203/rs.3.rs-6972996/v1

**Published:** 2025-07-02

**Authors:** Candido Faiela, Troy D Moon, Gustavo Amorim, Mohsin Sidat, Esperança Sevene

**Affiliations:** Department of Biological Science, Faculty of Science, Eduardo Mondlane University, Maputo, Mozambique; Department of Tropical Medicine and Infectious Diseases, Tulane University Celia Scott Weatherhead School of Public Health and Tropical Medicine, New Orleans, United States of America; Department of Biostatistics, Vanderbilt University Medical Center, Nashville, Tennessee, United States of America; Department of Community Health, Faculty of Medicine, Eduardo Mondlane University, Maputo, Mozambique; Department of Physiological Science, Faculty of Medicine, Eduardo Mondlane University, Maputo, Mozambique

**Keywords:** Antibiotics, Clinical decision support algorithm, Implementation Science, Upper respiratory tract infections, HIV, Mozambique

## Abstract

**Background:**

Antibiotics are widely overprescribed to treat upper respiratory tract infections (URTIs), even though viruses cause most URTIs. We evaluated the effectiveness of a clinical decision support algorithm (CDSA)- based intervention in reducing antibiotic prescriptions among ambulatory HIV-infected adult patients with acute URTI symptoms.

**Methods:**

Between June and September 2024, we conducted a multicenter, two-arm parallel, cluster-randomized controlled trial in six primary healthcare facilities in Mozambique. The intervention included applying the CDSA, educating and supervising clinicians, and conducting prescription audits. We used Pearson’s chi-square test and relative risk to assess the effectiveness of the intervention in reducing antibiotic prescribing.

**Results:**

Three hundred seventy-nine (97.9%) HIV-infected adult patients with URTI symptoms were recruited, 182 (48%) in the intervention arm and 197 (52%) in the control. Most were females (75.5%) and single (57%). Most appeared with common cold and flu-like symptoms. Participants in the intervention arm were less likely to receive an antibiotic prescription (RR 0.41, 95% CI: 0.31 – 0.55) and develop a complication (RR 0.44, 95% CI: 0.16 – 1.20) than those not exposed. The antibiotic prescribing rate was 23.1% for the intervention and 56.3% for the control. The intervention was associated with a significant reduction in antibiotic prescribing by 33.2% (p < 0.001) and a non-significant decrease in incidence of complications by 3.7% (p = 0.096). In both arms, most patients (78%) recovered completely within five days. Amoxicillin (47.8%), azithromycin (21.9%), and phenoxymethylpenicillin (14.1%) were the most prescribed antibiotics.

**Conclusions:**

Our CDSA, coupled with education and audits with feedback, effectively reduced antibiotic usage. Furthermore, withholding antibiotics for URTIs did not increase the incidence of complications. The intervention worked in our six sites, but larger studies must be performed with our CDSA across Mozambique to see if these findings also hold up elsewhere.

**Trial registration:**

ISRCTN, ISRCTN88272350. Registered 16 May 2024, https://www.isrctn.com/ISRCTN88272350

## Background

Despite extensive research over the years, the overuse and inappropriate prescribing of antibiotics for upper respiratory tract infections (URTIs) remains widespread [1]. Approximately 90% of URTIs are viral in origin, self-limiting, and typically resolve without complications, making antibiotic treatment unnecessary and not recommended [2,3]. Prescribing antibiotics in such cases is considered both wasteful and unwarranted [4]. Moreover, the misuse of antibiotics for URTIs can contribute to the development and spread of antibiotic-resistant organisms, ultimately making future infections more difficult to treat [5].

Opportunistic infections are the primary reason for prescribing antibiotics in adult HIV-infected patients, who require lifelong antiretroviral therapy (ART). Avoiding unnecessary antibiotic use in these patients can help minimize the risk of drug interactions and ultimately, adverse events [6, 7]. Furthermore, immunocompromised patients are at higher risk of adverse outcomes from unnecessary antibiotic use, including *Clostridioides difficile* infection [8].

Many studies have evaluated the use of clinical decision support algorithms (CDSA) to reduce antibiotic prescribing for URTIs in the context of primary care, with mixed results. Using a cluster randomized controlled trial design, two studies tried to evaluate the implementation of a particular CDSA in reducing or improving antibiotic prescribing for URTIs, but found no significant differences between the intervention and control arms [1,9]. Others, using a pre- and post-intervention and a randomized controlled trial design, tried to do the same (i.e., evaluating the implementation of a CDSA in decreasing or improving antibiotic prescribing), but found a significant difference favoring the intervention. May et al. (2021) reported a 12.6% decrease in inappropriate antibiotic prescriptions from the pre- to post-intervention period, while Rambaud-Athaus et al. (2017) found a drop in the antibiotic prescription rate from 70% in the control to 26% and 25%, respectively, for a CDSA in paper format or electronic format [10,11].

In Mozambique, the management of URTIs among HIV-infected patients in primary healthcare settings is predominantly empirical and often ends up with a prescription of antibiotics. In our previous work examining the types of prescriptions given to HIV-infected patients seen in an outpatient setting, roughly two-thirds (65.9%) of the patients received a prescription for antibiotics, most of them for respiratory tract infections [12]. Recommendations that came out of this work included developing strategies to promote the reduction of unnecessary antibiotic use in this population. Furthermore, these results informed the development of a larger multi-phase implementation science study protocol aimed at determining the effectiveness of a CDSA-based intervention in reducing unnecessary antibiotic prescriptions for URTIs among ambulatory HIV-infected adults. In this trial, we hypothesized that the CDSA, when introduced into routine outpatient care, would effectively reduce unnecessary antibiotic prescriptions by at least 15% [13].

## Methods

This analysis represents phase two data collection of our larger multi-phase implementation science study. Our study protocol has been published elsewhere [13]. The trial was registered on isrctn.com (ISRCTN88272350), a publicly accessible clinical trial registry recognized by the World Health Organization (WHO) and the International Committee of Medical Journal Editors (ICMJE). The study adheres to CONSORT 2025 guidelines.

### Study design

We conducted a multicenter two-arm parallel cluster randomized control trial design in which our primary outcome measure was the clinical decision to use antibiotics or not. Intervention elements included health worker education and supervision, prescription audit and feedback, organizational adjustments, and the introduction of a CDSA for decision-making around antibiotic use among ambulatory HIV-infected patients presenting with a URTI. The study tools were adapted before the intervention was implemented. An implementation audit and continuous feedback were performed to guarantee and monitor adherence to the intervention protocol.

### Study setting

This study was conducted within outpatient primary healthcare facilities in Maputo and Matola cities in Mozambique. Maputo is the nation’s capital, and Matola is the capital of Maputo Province, located around 20 km outside Maputo city. These primary healthcare facilities offer outpatient care for all ages, including HIV care and treatment clinics, family planning, maternal and child health services, youth and adolescent care, health counseling and screening, and an immunization program. All primary healthcare facilities within the study area were eligible for inclusion and considered for randomization. Six of the thirty-one eligible facilities in the catchment area were randomly selected for this study - four from Maputo and two from Matola.

### Randomization

Randomization and allocation were conducted at the level of 10 administrative units, referred to as primary clusters - municipal districts in Maputo and administrative posts in Matola. A random sequence was generated through the computer to assign six of these primary clusters equally to either the intervention or control arms (three each). Within each selected primary cluster, one primary healthcare facility (secondary cluster) was then randomly chosen to participate in the study. All participants within a given facility received the same group assignment. Due to resource constraints, only six primary clusters were included, and to prevent contamination between facilities, only one health facility was selected per cluster. The allocation sequence was generated by a statistician, who also assigned the selected facilities to either the intervention or control arm.

### Intervention and control

Eligible participants were adult HIV-infected individuals who presented to the outpatient clinics with symptoms of an acute URTI, such as nasal discharge or congestion, sore throat, cough, sneezing, chills, or disturbances in smell and taste, with or without fever. Patients were excluded if they exhibited symptoms of lower respiratory tract infection, had a fever of ≥ 39°C, severe mental illness, or advanced HIV disease.

Eligible patients allocated to the intervention arm were managed using the CDSA **(Supplementary File 1)**. Per the CDSA, patients were not to receive antibiotics if their URTI symptoms lasted less than 10 days, unless there was an additional symptom suggesting a suspicion of bacterial infection. Bacterial infection was suspected in the following situations: (i) higher-grade fever than usually observed with the common cold, with the presence of yellow or greenish nasal discharge, pain or difficulty swallowing, or an intense sore throat; (ii) URTI symptoms lasting longer than 10 days; (iii) URTI symptoms continuing to get worse rather than improve over several days (5 days after the first visit).

Twice per month, the study coordinator at each intervention site would review all antibiotic prescriptions provided to participants in the prior two weeks. Clinicians were provided feedback on the use of the CDSA in all cases where the CDSA was not followed and where no clinically appropriate justification was provided.

In the control sites, clinicians were instructed to continue managing patients according to their usual practices. Twice per month, antibiotic prescribing data from these clinicians were recorded, but no feedback was shared with them during the study period.

For both arms, patients were enrolled in the study at the time they presented to the clinic with URTI symptoms (t0 = day 0). They were then subsequently monitored through a phone call at three different time points after the initial medical visit to ascertain improvement of symptoms (t1 = day 5, t2 = day 10, and t3 = day 15). Participants were instructed that they could visit the healthcare facility for a follow-up clinical examination in person, at any time, if necessary.

### Data collection and measures

We used a structured questionnaire to collect patient-related sociodemographic and clinical information. We also utilized a case record notebook where additional information had been registered. The on-site coordinator was responsible for collecting data in synchrony with the clinicians. Twice per month, data quality checks were performed by the study PI for completeness, accuracy, consistency, validity, and uniqueness.

The effectiveness of the CDSA was analyzed by comparing antibiotic prescribing rates, incidence of complications, and mean time for complete recovery between the intervention and control arms. Our primary outcome was the antibiotic prescribing rate. Secondary outcomes consisted of the incidence of complications and the time for complete recovery from the first medical visit were the secondary outcomes. Complications were defined as worsening of symptoms that were deemed to result from not receiving antibiotics, such as sinusitis, pharyngotonsillitis, pneumonia, bronchitis, and asthma. These were documented for the first time at one of the follow-up visits. The antibiotic prescribing rate was calculated as the number of patients who received at least one antibiotic prescription on Day 0, among all participants enrolled. Antibiotics were classified according to the spectrum of action, prescription level according to the national medicine formulary (level 1, can be prescribed by a clinical agent/nurse; level 2, prescribed by a clinical technician; level 3, prescribed by a general practitioner; and level 4, prescribed by medical specialist), and WHO AWaRe 2023 classification. We included the AWaRe classification to check for a prescribing trend of antibiotics with a safety profile regarding adverse effects and the potential risk of resistance development. According to the AWaRe classification, antibiotics are classified as Access, Watch, and Reserve, taking into account the impact of different antibiotics on antimicrobial resistance [14]. The incidence of complications was measured as the proportion of complications among patients who were recruited and completed at least one follow-up visit. The mean time to complete recovery was calculated as the average time the patients took to recover completely from their symptoms.

### Data management, statistical analysis, and power calculation

Data collection forms and other patient registries were created in both paper and electronic format in REDCap (Research Electronic Data Capture) [15]. Study clinicians filled out the data collection forms during the initial visit, while the on-site study coordinator completed the forms during follow-up visits. These were then submitted to the study PI, who input data from the forms into the REDCap system. Data were then exported to SPSS (Statistical Package for Social Sciences) version 25 for statistical analysis [15, 16].

Descriptive and analytical statistics were used to analyze the data. Descriptive analysis involved constructing tables of absolute and relative frequencies. Analytical statistics was used for bivariate analysis. Pearson’s chi-square test was employed to compare outcomes between intervention and control sites, using a 5% significance level. The same test was used to assess baseline comparability between the two arms and to evaluate differences in recovery time rates. To quantify the intervention’s impact, relative risk (RR) was calculated, along with the effectiveness ratio (1 – RR), and 95% confidence intervals were reported for RR.

Sample sizes were selected based on power calculations to detect a minimum difference of 15% in antibiotic prescribing rates between the two arms, with 80% power and type I error set at 5%.

## Results

### Participants

**Figure 1:**
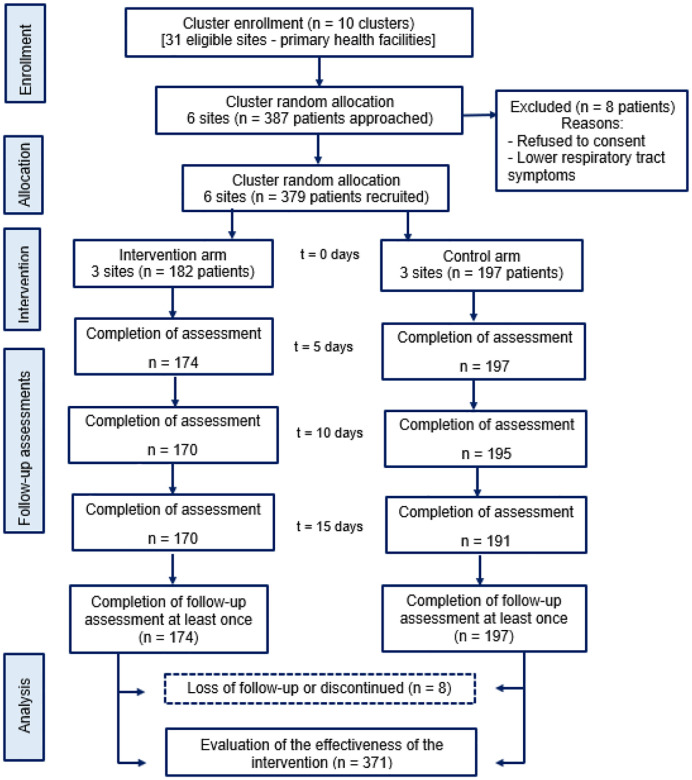
Study flow diagram: enrollment, intervention, and assessments

Among 387 patients approached, 379 (97.9%) were successfully recruited into the study. Two patients (0.5%) were excluded from enrollment due to their refusal to provide consent, and 6 (1.6%) were excluded as they were subsequently determined to have a lower respiratory tract infection. Of the patients recruited, 182 (48%) were allocated to the intervention arm and 197 (52%) to the control arm. Of these, 174 (95.6%) patients in the intervention arm and 197 (100%) patients in the control arm participated in at least one follow-up visit (Fig. 1).

The study participants were adults aged 18 years or older, with a mean age of 44 years (SD = 12.3), and a majority were female (75.5%). Over half of the participants (57%) were single and had a secondary level of education or higher (59.9%) (Table 1). The majority of participants presented to clinic with common cold and flu-like symptoms, with no fever (84.4%) or only a low-grade fever (13%), a cough lasting less than 10 days (74.9%), headache (60.2%), rhinorrhea (59.6%), nasal congestion lasting less than 10 days (53.8%), and sore throat (49.1%). Of our enrolled participants, roughly two-thirds (65.8%) were enrolled at clinics in the city of Maputo.

When comparing sociodemographic characteristics of the participants in the intervention arm versus the control arm, we found no major differences except for a statistically significantly higher proportion of control participants with a secondary education or higher (66.9% vs. 52.5%, p < 0.001). Additionally, a higher proportion of participants in the control arm presented with a low-grade fever (29.7% vs 6.6%) or a high-grade fever (4.7% vs. 1.8%) (p < 0.001) as compared to those in the intervention arm. As part of the study, each intervention site received at least one thermometer for use during the study. As such, a much larger proportion of participants in the intervention arm had their body temperature recorded than in the control arm (91.7% vs. 32.4%).

### Antibiotic prescribing rates

**Figure 2:**
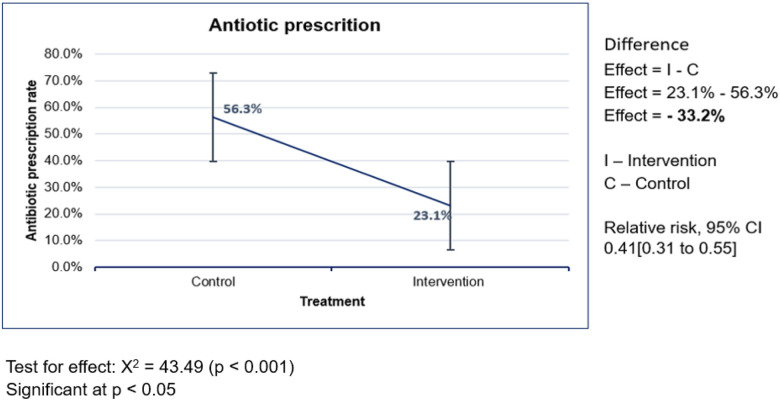
Comparison of antibiotic prescribing rates between intervention and control arms on the day of recruitment (Day 0). A reduced frequency is observed in the intervention arm. Pearson’s chi-square test was used to test the significance of the reduction (p < 0.001). RR was less than 1, meaning that the intervention had a protective effect.

Overall, 40.4% of ambulatory HIV-infected patients presenting with a URTI were prescribed an antibiotic. When comparing study arms, antibiotic prescribing was higher in the control group (56.3%) compared to the intervention group (23.1%), representing a 33.2% reduction in antibiotic prescribing (p < 0.001) (Fig. 2). Patients managed with the CDSA (intervention arm) had a 59% lower likelihood of antibiotic prescription compared to the control group (RR = 0.41; 95% CI: 0.31–0.55).

### Antibiotic prescribing pattern

In a review of medications prescribed throughout the study, we found that nine types of antibiotics were among those most prescribed, of which eight were broad-spectrum. Amoxicillin (47.8%), azithromycin (21.9%), and phenoxymethylpenicillin (14.1%) were the most prescribed. Six of the nine antibiotics are classified as “*access*” antibiotics and three as “*watch*” antibiotics according to the AWaRe classification. Among the most prescribed antibiotics, amoxicillin and phenoxymethylpenicillin are classified as “*access*” and azithromycin as “*watch*” (Table 3). Regarding the prescription level according to the national medicine formulary, four were level 1 antibiotics, two were level 2, and three were level 3.

### Incidence of complications

**Figure 3:**
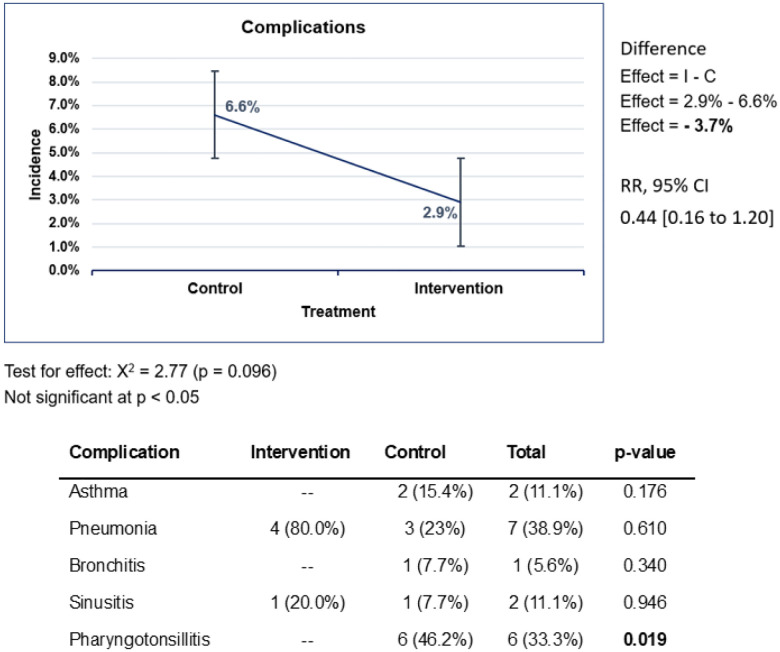
Comparison of the incidence of complications between intervention and control arms. A reduced incidence of complications is observed in the intervention arm. Pearson’s chi-square test was used to test the significance of the reduction (*p* = 0.096). Although the reduction was insignificant, the RR was less than 1, meaning that the intervention reduced the risk of developing a complication.

We attempted to follow participants in both arms for 15 days following their initial clinic visit, with reassessments scheduled for days 5, 10, and 15 to assess for any complications (Fig. 3). When comparing study arms, the rate of complications was higher in the control group (6.6%) compared to the intervention group (2.9%), representing a 3.7% reduction in complications seen (p = 0.096) (Fig. 3). Patients managed with the CDSA (intervention arm) had a 56% lower likelihood of developing a complication as compared to the control group (RR = 0.44; 95% CI: 0.16–1.20), though this was not statistically significant. The most frequently observed complications were pneumonia (intervention arm 80% vs. control arm 23%) and pharyngotonsillitis (control arm only 46.2%).

### Time to complete recovery from the first medical visit

**Figure 4:**
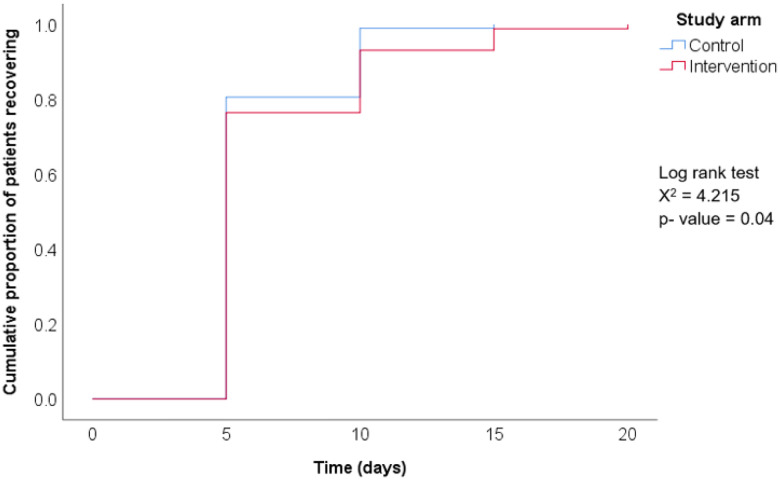
Cumulative probability of patients recovering stratified by control and intervention arms. Irrespective of study arms, all participants are fully recovered in about 2 weeks of symptom onset. Log-rank test was used to test for equality of recovery distributions between the two curves (i.e., intervention and control). Statistically significant differences were observed around days 10 and 15 of follow-up visits, favoring control, but with no clinical significance.

Table 4 shows the cumulative data stratified by study group and over 20 days. Complete recovery was seen in most participants (78%) within five days of their initial visit, regardless of study arm, with a mean time to complete recovery of 6.3 days (SD = 2.7). No significant differences (p = 0.378) were observed between the intervention and control arms regarding time to recovery within five days. Figure 4 shows the cumulative probability of patients recovering by study arm. The control arm seems to recover slightly faster than the intervention group. Still, this difference is not clinically significant, as regardless of the study arms, all participants fully recovered in about 2 weeks of symptom onset or treatment.

## Discussion

This trial demonstrated the effectiveness of a CDSA-based intervention in reducing antibiotic prescribing for URTIs among HIV-infected adults in the primary healthcare settings. Clinician use of the CDSA tool - coupled with targeted education, prescription audits, and feedback - was associated with a 33.2% reduction in antibiotic use compared to the control arm. This reduction was more than double the anticipated effect size of 15% specified in our implementation protocol [13].

Participants in the intervention arm were significantly less likely to receive an antibiotic prescription than those in the control arm (RR = 0.41; 95% CI: 0.31–0.55). This reduction is attributed to clinicians’ commitment to de-implementing unnecessary antibiotic prescribing practices. Notably, the antibiotic prescribing rate in the intervention arm (23.1%) fell within the WHO-recommended reference range of 20–26.8%, underscoring the value of using the CDSA to guide rational antibiotic use within this population [17, 18].

In contrast, the control arm had a higher antibiotic prescribing rate (56.3%), exceeding WHO recommendations, though still lower than the baseline level (65%). This reduction from baseline may be partially due to clinicians’ exposure during the pre-implementation phase, where they became aware of the study’s aims and may have modified their prescribing behavior accordingly. Additionally, the difference in data collection methods—retrospective for baseline and prospective for the implementation phase—could have influenced clinician behavior. Participants in prospective studies often modify their actions due to the perception of being observed, a known phenomenon that can lead to improved prescribing practices [19]. Our findings align with a study from Tanzania that reported a 26% antibiotic use rate in the CDSA group versus a 70% rate in the control group [11].

Overall, the combined antibiotic prescribing rate in our study (40.4%) was lower than rates reported in other studies in Mozambique—97.6% by Monteiro et al. (2017); 97.5% by Xavier et al. (2022) among pediatric patients; and 65.9% by Faiela & Sevene (2022) among HIV-infected adults. It was also lower than rates reported in neighboring countries—84.9% in Tanzania and 70.6% in Botswana [12,20,21,22,23]. However, our findings are consistent with reports from South Africa (37.7%) and Kenya (46.7%) [24,25]. The reduced rate observed in this study is attributed to the CDSA, which helped clinicians manage URTIs with a rational clinical decision, including de-implementing unnecessary antibiotics and only prescribing them if a bacterial infection was suspected. In addition to the CDSA, we feel the presence of an on-site coordinator who monitored and supervised the study’s implementation process, reminding clinicians (in the intervention arm) to adhere to the CDSA and conducting prescription audits with feedback to the clinicians, contributed to this reduction.

When antibiotics were prescribed, amoxicillin (48%) and phenoxymethylpenicillin (14%) were the most commonly used, consistent with first-line recommendations for bacterial URTIs like tonsillitis and pharyngitis [26,27]. However, the use of azithromycin (22%) was unexpectedly high, given that it is typically reserved for patients with penicillin allergies. This trend is concerning due to its higher cost and the potential for fostering antimicrobial resistance. Although azithromycin was widely used during the COVID-19 pandemic, there is no evidence to support its use for viral URTIs [28].

Despite established guidelines advising against routine antibiotic use for URTIs, some clinicians prescribe them to prevent complications [29]. However, our findings showed no increased risk of complications in the intervention arm. There was a non-significant 3.7% reduction in complication rates (p = 0.096) compared to the control arm. Pneumonia (38.9%) and pharyngotonsillitis (33.3%) were the most common complications.

Patient recovery within five days was slightly higher in the control arm, where over half received antibiotics. However, more than three-quarters of patients in the intervention arm also recovered within five days, despite lower antibiotic use, highlighting one measure of the intervention’s effectiveness. Since bacterial URTIs may take several days to resolve, many patients in the control group likely received unnecessary antibiotics [30,31].

Most patients presented with mild symptoms, such as the common cold and flu-like illness. Empiric antibiotic therapy should be reserved for patients with suspected bacterial infection (i.e., high-grade fever, purulent nasal discharge, difficulty swallowing, or persistent worsening symptoms), particularly when the risk of complications is high [13,32]. Broader implementation of this CDSA-based intervention may further reduce unnecessary antibiotic use, helping mitigate stockouts, reduce treatment costs, and slow the development of antimicrobial resistance [33].

This study has several limitations. The trial was conducted in only six healthcare facilities selected through convenience sampling, which may limit generalizability. Contamination between study arms may have occurred due to earlier engagement during the pre-implementation and adaptation phases. Recruitment rates differed slightly between arms but did not affect the overall interpretation of results.

## Conclusions

Our CDSA, coupled with education and audits with feedback, effectively reduced antibiotic usage. Furthermore, when decisions were made to withhold antibiotics for URTI, this approach did not increase the incidence of new symptoms or complications. In our six study sites, the intervention worked, but larger studies need to be performed with this CDSA across Mozambique to see if these results also hold up elsewhere. Thus, advocacy should be maintained with the Ministry of Health to implement this CDSA-based intervention in larger sites through an implementation science approach using frameworks that can measure effectiveness outcomes.

## Supplementary Material

Supplementary Files

This is a list of supplementary files associated with this preprint. Click to download.

- Supplementaryfile1.docx
- DatabaseE2v3FINAL27JUNE20251.sav

## Figures and Tables

**Table 1:** Sociodemographic and clinical characteristics of the study participants

Characteristic (n = 379)	Intervention	Control	Total	p-value
Sex				0.875
Male	44 (24.2%)	49 (24.9%)	93 (24.5%)	
Female	138 (75.8%)	148 (75.1%)	286 (75.5%)	
Age				0.051
18–35 years	35 (19.2%)	60 (30.5%)	95 (25.1%)	
36–45 years	65 (35.7%)	51 (25.9%)	116 (30.6%)	
46–59 years	62 (34.1%)	65 (32.9%)	127 (33.5%)	
≥ 60 years	20 (11.0%)	21 (10.7%)	41 (10.8%)	
Marital Status				0.276
Married	67 (36.8%)	70 (35.5%)	137 (36.6%)	
Divorced	6 (3.3%)	4 (2.0%)	10 (2.7%)	
Single	106 (58.2%)	113 (57.4%)	219 (57.0%)	
Widower	3 (1.6%)	10 (5.1%)	13 (3.7%)	
Level of Education (n = 361)				< 0.001
Illiterate	6 (3.4%)	14 (7.6%)	20 (5.5%)	
Primary	78 (44.1%)	47 (25.5%)	125 (34.6%)	
Secondary/technical	88 (49.7%)	105 (57.1%)	193 (53.5%)	
Higher	5 (2.8%)	18 (9.8%)	23 (6.4%)	
Fever(n = 231)				< 0.001
Low grade	11 (6.6%)	19 (29.7%)	30 (13%)	
High grade	3 (1.8%)	3 (4.7%)	6 (2.6%)	
No fever	153 (91.6%)	42 (65.6%)	195 (84.4%)	
Clinical signs/symptoms				
Rhinorrhea	113 (62.1%)	113 (57.4%)	226 (59.6%)	0.349
Sore throat	80 (44%)	106 (53.8%)	186 (49.1%)	0.055
Cough < 10 days	139 (76.4%)	145 (73.6%)	284 (74.9%)	0.534
Cough > 10 days	11 (6%)	12 (6.1%)	23 (6.1%)	0.985
Nasal congestion < 10 days	89 (48.9%)	115 (58.4%)	204 (53.8%)	0.065
Nasal congestion > 10 days	5 (2.7%)	5 (2.5%)	10 (2.6%)	0.899
Chills	66 (36.3%)	67 (34%)	133 (35.1%)	0.646
Runny nose	61 (33.5%)	45 (22.8%)	106 (28%)	0.021
Headache	114 (62.6%)	117 (57.9%)	227 (60.2%)	0.329
Place of Study (n = 377)				0.516
Maputo	112 (64.0%)	136 (67.3%)	248 (65.8%)	
Matola	63 (36.0%)	66 (32.7%)	129 (34.2%)	

**Table 3:** Antibiotic prescribing patterns

Antibiotic name	Class	Spectrum of action	Prescription level*	AwaRe 2023	Day 0 (recruitment visit)	Day 5 (1st follow-up visit)	Day 10 (2nd follow-up visit)	Day15 (3rd follow-up visit)	Total
Amoxicillin	Penicillin	Broad	1	Access	88 (51.5%)	9 (32.1%)	1 (25.0%)	----	98 (47.8%)
Amoxicillin-clavulanic acid	Beta-lactamase inhibitor	Broad	3	Access	3 (1.8%)	3 (10.7%)	1 (25.0%)	----	7 (3.4%)
Azithromycin	Macrolides	Broad	3	Watch	39 (22.8%)	4 (14.3%)	1 (25.0%)	1 (50.0%)	45 (21.9%)
Cefixime	Third-generation-cephalosporins	Broad	3	Watch	4 (2.3%)	2 (7.1%)	----	----	6 (2.9%)
Chloramphenicol	Amphenicols	Broad	2	Access	----	----	----	1 (50.0%)	1 (0.5%)
Cotrimoxazole	Sulfonamide	Broad	1	Access	10 (5.8%)	4 (14.3%)	---	----	14 (6.8%)
Erythromycin	Macrolides	Broad	2	Watch	2 (1.2%)	----	----	----	2 (1.0%)
Metronidazole	Imidazole	Broad	1	Access	2 (1.2%)	1 (3.6%)	----	----	3 (1.5%)
Phenoxymethylpenicillin	Penicillin	Narrow	1	Access	23 (13.4%)	5 (17.9%)	1 (25.0%)	----	29 (14.1%)

* Prescription level according to Mozambique National Medicines Formulary 2007

**Table 4:** Time to complete recovery between the two treatment arms.

Time to recovery	Intervention	Control	Total	*p-value*
	N (%) *	N (%) *	N (%) *	
Day 5	133 (76%)	158 (80%)	291 (78%)	0.378
Day 10	162 (93%)	195 (99%)	357 (96%)	**0.010**
Day 15	172 (99%)	197 (100%)	369 (99%)	0.488
Day 20	174 (100%)	197 (100%)	371 (100%)	---
Mean ± SD	6.5 ± 3.2	6.0 ± 2.2	6.3 ± 2.7	

* Accumulated frequency

## Data Availability

Data not publicly available. All generated data will be available from the corresponding authors upon reasonable request and are deposited at the Faculty of Medicine, University Eduardo Mondlane data repository with limited access.

## Abbreviations

ARV: Antiretroviral
AWaRe: Access, watch, and reserve the WHO antibiotic classification system
CDSA: Clinical decision support algorithm
CI: Confidence interval
HIV: Human immunodeficiency virus
PI: Principal investigator
RCT: Randomized controlled trial
REDCap: Research electronic data capture
RR: Relative risk
SD: Standard deviation
SPSS: Statistical package for social sciences
URTI: Upper respiratory tract infection
WHO: World Health Organization
